# Assessment of Genetic Diversity and Population Genetic Structure of *Corylus mandshurica* in China Using SSR Markers

**DOI:** 10.1371/journal.pone.0137528

**Published:** 2015-09-10

**Authors:** Jian-Wei Zong, Tian-Tian Zhao, Qing-Hua Ma, Li-Song Liang, Gui-Xi Wang

**Affiliations:** 1 State Key Laboratory of Tree Genetic and Breeding, Research Institute of Forestry, Chinese Academy of Forestry, Beijing, China; 2 College of Resource and Environmental Science, Pingdingshan University, Pingdingshan, Henan Province, China; Beijing Forestry University, CHINA

## Abstract

*Corylus mandshurica*, also known as pilose hazelnut, is an economically and ecologically important species in China. In this study, ten polymorphic simple sequence repeat (SSR) markers were applied to evaluate the genetic diversity and population structure of 348 *C*. *mandshurica* individuals among 12 populations in China. The SSR markers expressed a relatively high level of genetic diversity (*Na* = 15.3, *Ne* = 5.6604, *I* = 1.8853, *Ho* = 0.6668, and *He* = 0.7777). According to the coefficient of genetic differentiation (*F*_*st*_ = 0.1215), genetic variation within the populations (87.85%) were remarkably higher than among populations (12.15%). The average gene flow (*Nm* = 1.8080) significantly impacts the genetic structure of *C*. *mandshurica* populations. The relatively high gene flow (*Nm* = 1.8080) among wild *C*. *mandshurica* may be caused by wind-pollinated flowers, highly nutritious seeds and self-incompatible mating system. The UPGMA (unweighted pair group method of arithmetic averages) dendrogram was divided into two main clusters. Moreover, the results of STRUCTURE analysis suggested that *C*. *mandshurica* populations fell into two main clusters. Comparison of the UPGMA dendrogram and the Bayesian STRUCTURE analysis showed general agreement between the population subdivisions and the genetic relationships among populations of *C*. *mandshurica*. Group I accessions were located in Northeast China, while Group II accessions were in North China. It is worth noting that a number of genetically similar populations were located in the same geographic region. The results further showed that there was obvious genetic differentiation among populations from Northeast China to North China. Results from the Mantel test showed a weak but still significant positive correlation between Nei’s genetic distance and geographic distance (km) among populations (r = 0.419, *P* = 0.005), suggesting that genetic differentiation in the 12 *C*. *mandshurica* populations might be related to geographic distance. These data provide comprehensive information for the development of conservation strategies of these valuable hazelnut resources.

## Introduction

Hazelnut, *Corylus mandshurica* Maxim. et Rupr (synonym to *C*. *sieboldiana*), belongs to the family Betulaceae and is an important species, both economically and ecologically, among nut trees. *C*. *mandshurica* is a deciduous shrub of about 2 to 6 m in height with bracts forming a tubular husk [1–3].The leaves are irregularly serrate and ovate leaves alternate with circular leaves. Their abaxial surface is covered heavily with pubescence. *C*. *mandshurica* is widely distributed in Japan, Korea, and Northeast to North China around Beijing. Most of *C*. *mandshurica* survived in the mountainous forest belts and deep valleys at high altitude [4]. The shells are thin and thus the ratio of kernel weight to nut weight is high [5], and the nuts are rich in nutrients, mainly including unsaturated fat, protein and vitamins [6]. Compared with *C*. *avellana*, the nuts have better taste and fragrance. Its nuts are not only suitable for fresh eating, but also suitable for baking. Its nuts are an important ingredient in cake, and are a favorite of consumers [7]. Moreover, its leaves can be used as fodder to feed domestic silkworms by local farmers. In addition to its economic value, hazelnut is useful for soil and water conservation and sustainable use of local forests [8].

In recent years, molecular markers have shown promise for assessment of genetic diversity, owing to their high discriminatory power and comparatively low cost. In particular, the superiority of molecular markers for the characterization of plants is well recognized. DNA based molecular marker technologies, such as simple sequence repeat (SSR), amplified fragment length polymorphism (AFLP), sequence-related amplified polymorphism (SRAP) and single nucleotide polymorphisms (SNPs), have several advantages including abundant, independent from the environment, suitability for early and rapid evaluation, and having non-tissue specific characteristics. Among them, SSRs, also known as microsatellites or short tandem repeats (STR), are widely present in eukaryotic genomes [9] and very useful for a number of reasons including co-dominant inheritance, high polymorphism, high variability and suitability for automated allele sizing and cross-species transferability.

SSRs have been developed for European hazelnut [10–14], and parentage relationships among the most important hazelnut cultivars were identified [15]. The genetic linkage map has been established [16–18]. Moreover, microsatellite markers are considered as valuable tools in molecular breeding of *C*. *mandshurica*[19]. Besides, SSR markers have already been successfully applied to the evaluation of the genetic diversity and population structure of European hazelnut [20–21], comprising eastern filbert blight (EFB)-resistant cultivars [22]. Evaluation of genetic diversity by SSR markers is of importance not only for the breeding of hazelnut, but also for the efficient management and protection of hazelnut germplasm. Characterization of the genetic diversity and genetic differentiation is indispensable for the efficient protection of hazelnut by helping understand its population dynamics, origin and the evolution process [23]. However, limited information has been acquired on the genetic diversity of *C*. *mandshurica* resources in China, and its genetic variation and population genetic structure are still unknown.

In this study, we present the first investigation of genetic diversity of wide-ranging hazelnut (*C*. *mandshurica*) in China, with a particular focus on population structure using SSR markers. A total of 12 *C*. *mandshurica* populations throughout its present main Chinese distribution range were studied. The aims of this study were to: (1) evaluate the genetic diversity and population structure of *C*. *mandshurica*, (2) estimate variance components and partition the within- and among-population variance, (3) obtain comprehensive information for the development of conservation strategies for these valuable *C*. *mandshurica* resources.

## Materials and Methods

### Ethics statement

The collection of plant samples and research activities were conducted with the permission of local forestry departments. No other endangered or protected species were involved in this study.

### Population sampling and DNA extraction

**Fig 1:**
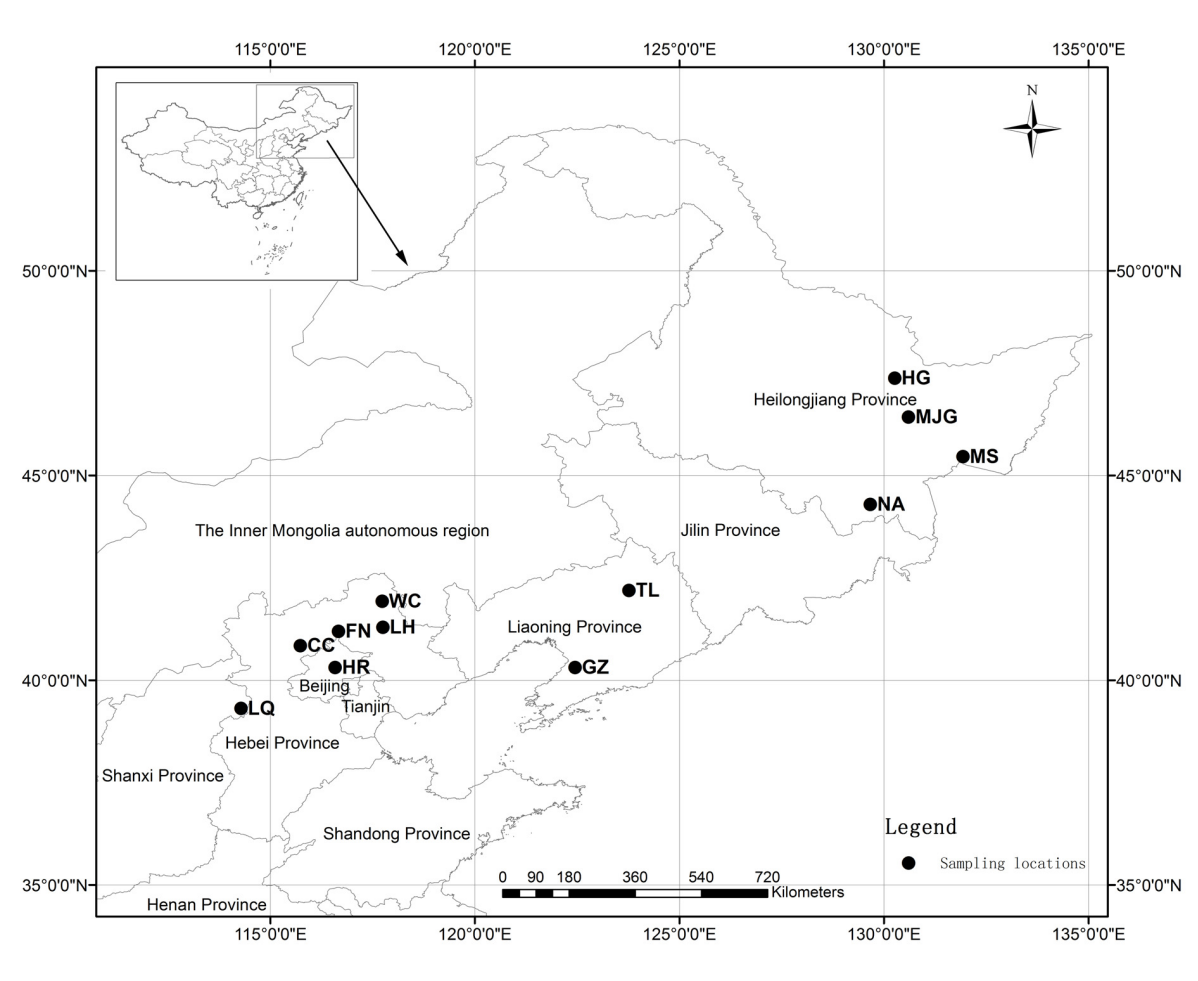
Geographic location of 12 *C*. *mandshurica* populations sampled in China. Population codes are identified in Table 1.

A total of 348 trees of *C*. *mandshurica* were collected from 12 populations, each represented by 20 to 31 individuals (located at least 100 m apart) (Fig 1, Table 1). Fresh young leaf tissues (three to five leaves per tree) of individual plants were collected and promptly dried with silica gel in a sealed manila envelope and then taken back to the laboratory for later DNA extraction. Total DNA was extracted from about 20 mg of leaf tissue per tree, using the modified CTAB procedure [24].

**Table 1:** Location of populations, number of individuals sampled in a study of genetic diversity of *Corylus mandshurica* in China by SSR analyses.

Population code	Location	Number of samples	Latitude (N)	Longitude (E)	Altitude (m)
MJG	Mengjiagang, Heilongjiang	30	46°26'	130°36'	326
HR	Huairou, Beijing	26	40°19'	116°35'	180
LH	Longhua, Hebei	28	41°18'	117°45'	643
LQ	Shangzai, Shanxi	31	39°19'	114°17'	1391
FN	Xiamiao, Hebei	29	41°12'	116°40'	723
NA	Ning'an, Heilongjiang	30	44°18'	129°40'	534
MS	Mishan, Heilongjiang	20	45°28'	131°56'	146
WC	Weichang, Hebei	30	41°56'	117°44'	980
TL	Fanhe, Liaoning	30	42°12'	123°46'	125
CC	Chicheng, Hebei	33	40°51'	115°44'	1302
HG	Xinhua, Heilongjiang	31	47°23'	130°16'	214
GZ	Shagang, Liaoning	30	40°19'	122°27'	214
Total		348			

### Simple sequence repeat (SSR)

Ten microsatellite loci, CAC-A040, CAC-B005, CAC-B001, CAC-B020, CAC-B028, CAC-B113, CAC-B114, CAC-C003, CAC-C028, and CAC-B105 were analyzed for polymorphism [10, 25] (Table 2). Polymerase chain reaction (PCR) amplification was performed in a total of 20 μL volume that contained 10–50 ng of plant DNA, 0.2 mM each of forward and reverse primers, 1.5 mM MgCl_2_, 50 mM Tris–HCl, 0.2 mM of each dNTP, 1 unit of Taq DNA polymerase (Biotech International) and accompanying buffer. PCR amplification was performed with the following cycling parameters: first a denaturation step at 94°C for 5min, followed by 35 cycles of 94°C for 30 s, annealing at 48°C~55°C for 40 s (different primer annealing temperature,Table 2), 72°C for 40 s and a final extension at 72°C for 3 min. The forward primers were labeled with a fluorochrome (FAM). Amplified fragments of SSRs were analyzed with an ABI 3730XL capillary sequencer (Applied Biosystems) separately along with an internal size standard (GeneScan-500 LIZ, Applied Biosystems). The SSR allele sizes were called with GENEMAPPER software (version 4.0, Applied Biosystems) for all populations and entered in a spreadsheet.

**Table 2:** Genetic diversity at 10 SSR loci in 348 individuals of *C*. *mandshurica*.

Locus	Allele size range(bp)	Annealing temperature(°C)	*Na*	*Ne*	I	*Ho*	*He*
CAC-A040	250-300bp	50	13	3.2180	1.6361	0.5891	0.6902
CAC-B001	98-120bp	50	19	7.5308	2.2955	0.8928	0.8685
CAC-B114	140-160bp	55	17	5.9692	2.0581	0.5934	0.8337
CAC-C028	120-150bp	51	7	2.0804	0.9193	0.4339	0.5201
CAC-C003	100-130bp	48	10	3.7291	1.5314	0.4725	0.7329
CAC-B113	148-180bp	51	12	6.3050	2.0886	0.7666	0.8426
CAC-B105	130-160bp	51	16	5.8002	1.9360	0.7861	0.8288
CAC-B028	260-280bp	51	18	6.6402	2.1356	0.7420	0.8506
CAC-B020	220-270bp	50	28	12.1102	2.7178	0.8309	0.9188
CAC-B005	270-280bp	51	13	3.2209	1.5347	0.5607	0.6905
Mean			15.3	5.6604	1.8853	0.6668	0.7777
St. Dev			5.8128	2.8877	0.4990	0.1572	0.1188

For each locus: Number of alleles observed (*Na*), Effective number of alleles (*Ne*), Shannon's Information index (I), Observed heterozygosity (*Ho*), Expected heterozygosity (*He*), Gene flow (*Nm*)

### Data analysis

The presence of null alleles of each locus was estimated by software MICRO-CHECKER 2.2.3 [26]. The number of alleles observed (*Na*), effective number of alleles (*Ne*) [27], Shannon's information index (*I*), observed heterozygosity (*Ho*), expected heterozygosity (*He*), genetic differentiation coefficient (*F*_*st*_), gene flow (*Nm*) and Nei’s genetic distance (*Gd*) [28](S1 Table) were calculated using Popgene32 software. *Nm* was estimated as: *Nm* = 0.25(1−*F*_*st*_) / *F*_*st*_. Cluster analysis was carried out based upon the Nei’s genetic distance matrix with UPGMA (unweighted pair group method of arithmetic averages) and a dendrogram was constructed using MEGA (version 5) [29].

Genetic structure analysis was performed with the program STRUCTURE 2.3.4 with 10 runs and 100,000 Markov Chain Monte Carlo (MCMC) repetitions after a burn-in period of 100,000 interactions for each group number K. The optimum value of K was obtained by calculating the ∆k value to determine the most likely number of groups [30]. Analysis of molecular variance (AMOVA) was carried out using the program Arlequin (version 3.5) for estimation of variance components and partition of the within- and among-population variance [31]. In addition, the Mantel test was conducted using the program GenALEx 6.5 for correlation between Nei’s genetic distance [32] and geographic distance (km) [33]. Significance was assessed by conducting 9999 permutations.

## Results

### Microsatellite variation and genetic diversity

In this study, MICRO-CHECKER analysis indicated the presence of null alleles at a few loci in some populations. At CAC-B114, CAC-C003 and CAC-B028, null alleles were detected in five populations, at CAC-B105, CAC-A040, CAC-B020 and CAC-B005, null alleles were detected in two populations, and at CAC-C028, null alleles were detected in population TL, while at CAC-B001 and CAC-B113, no null alleles were detected. Thus, these 10 SSR loci are a proper set to assess genetic diversity of *C*. *mandshurica*.

A total of 153 alleles were found at the ten microsatellite loci in the 348 individuals (Table 2). The number of alleles observed (*Na*) per locus varied from 7 (CAC-C028) to 28 (CAC-B020) with a mean of 15.3 per locus. At the same time, effective numbers of alleles (*Ne*) **v**aried from 2.0804 (CAC-C028) to 12.1102 (CAC-B020) with an average of 5.6604 per locus. Shannon's Information index (*I*) averaged 1.8853 and ranged from 0.9193 (CAC-C028) to 2.7178 (CAC-B020). Observed heterozygosity (*Ho*) and expected heterozygosity (*He*) ranged from 0.4339 (CAC-C028) and 0.5201 (CAC-C028) to 0.8928 (CAC-B001) and 0.9188 (CAC-B020) respectively.

### Genetic differentiation

The inbreeding coefficient (*F*_*is*_) per locus ranged from -0.1167 (CAC-B001) to 0.1143 (CAC-B114), with an average of 0.0215 alleles per locus. Furthermore, genetic differentiation (*F*_*st*_) of individual loci ranged from 0.0689 at CAC-A040 to 0.2016 at CAC-B114, and averaged at a value of 0.1215 alleles per individual locus, suggesting low genetic differentiation among the populations. Moreover, the genetic diversity within populations (87.85%) was significantly higher than that between populations (12.15%). In addition, gene flow (*Nm*) ranged from 0.9903 at CAC-B114 to 3.3798 at CAC-A040 and averaged 1.8080 (Table 3). Similarly, the results of AMOVA analysis indicated that variation within populations was 88.50% while variation among populations was 11.50% (Table 4), possibly because of the relatively high gene flow (*Nm* = 1.8080) between *C*. *mandshurica* populations.

**Table 3:** Summary of F statistics and gene flow for the 10 loci.

Locus	*F*_*is*_	*F*_*st*_	*Nm*
CAC-A040	0.0935	0.0689	3.3798
CAC-B001	-0.1167	0.0770	2.9960
CAC-B114	0.1143	0.2016	0.9903
CAC-C028	0.0749	0.0878	2.5972
CAC-C003	0.2052	0.1772	1.1607
CAC-B113	-0.0616	0.1413	1.5195
CAC-B105	-0.0718	0.1113	1.9968
CAC-B028	0.0275	0.1055	2.1196
CAC-B020	0.0119	0.0834	2.7469
CAC-B005	0.0200	0.1579	1.3336
Mean	0.0215	0.1215	1.8080

For each locus: *F*_*is*_, coefficient of inbreeding; *F*_*st*_, Genetic differentiation coefficient; *Nm*, Gene flow

**Table 4:** Analysis of molecular variance (AMOVA) of genetic diversity of *C*. *mandshurica* populations.

Source of variation	d.f.	Sum of squares	Variance components	Percentage of variation	P
Among populations	11	317.662	0.43999	11.50%	<0.001
Within populations	684	2316.455	3.38663	88.50%	<0.001
Total	765	2947.051	3.89756		

### Genetic relationships and population structure analysis

**Fig 2:**
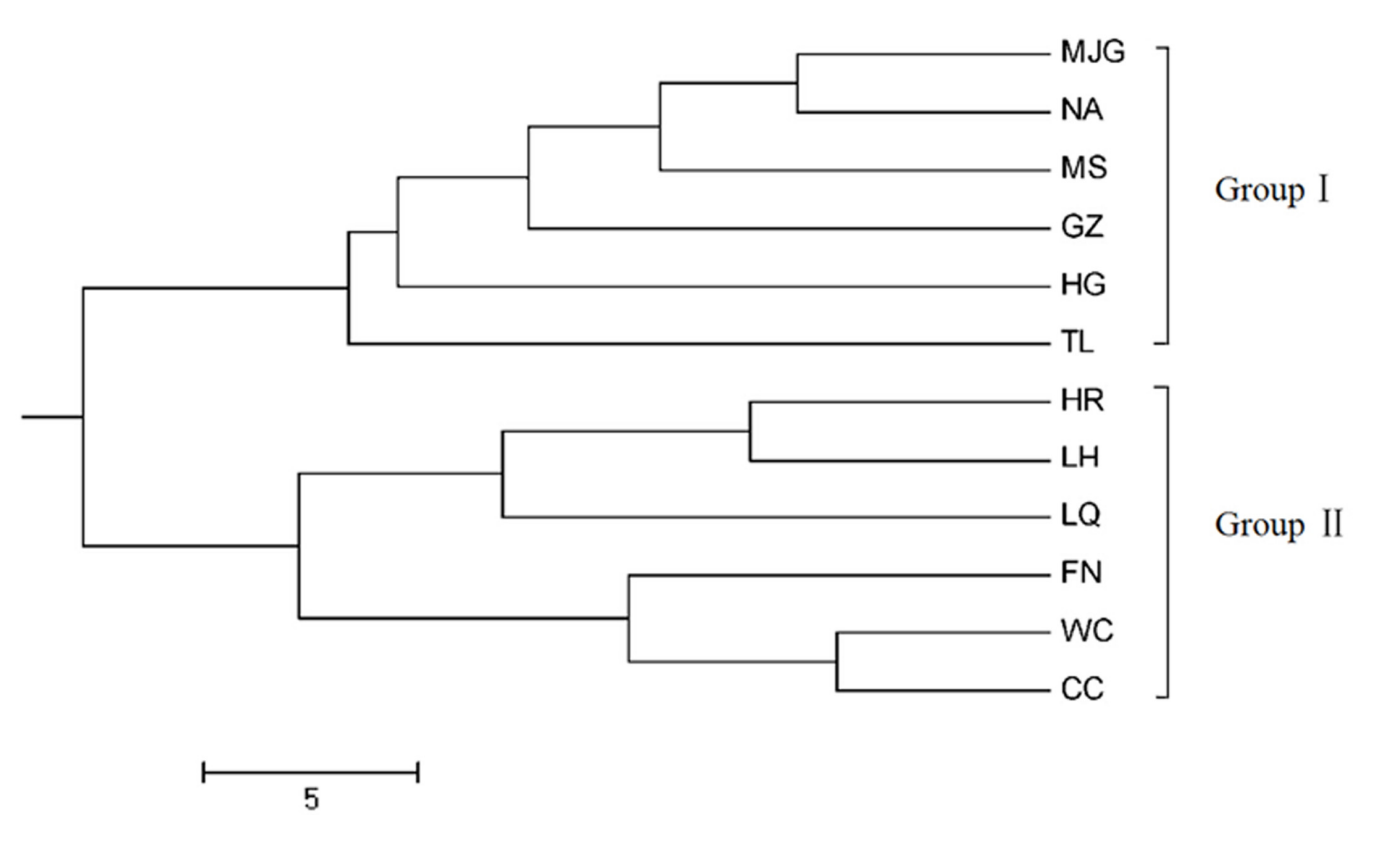
UPGMA dendrogram of 12 populations of *C*.*mandshurica* based on Nei's genetic distance at 10 SSR loci.

A dendrogram was drawn based on the SSR data depicting the genetic relationships among the 12 populations (Fig 2). The populations were differentiated into two main clusters, and each cluster was further separated into smaller clusters. Group I included populations from Heilongjiang Province (MJG, NA, MS and HG) and Liaoning Province (GZ and TL). Group II contained populations from Hebei Province (LH, FN, WC and CC), Shanxi Province (LQ) and Beijing City (HR), and was separated into two sub clusters. The results showed that distinct genetic differentiation among populations from Northeast China and North China.

**Fig 3:**
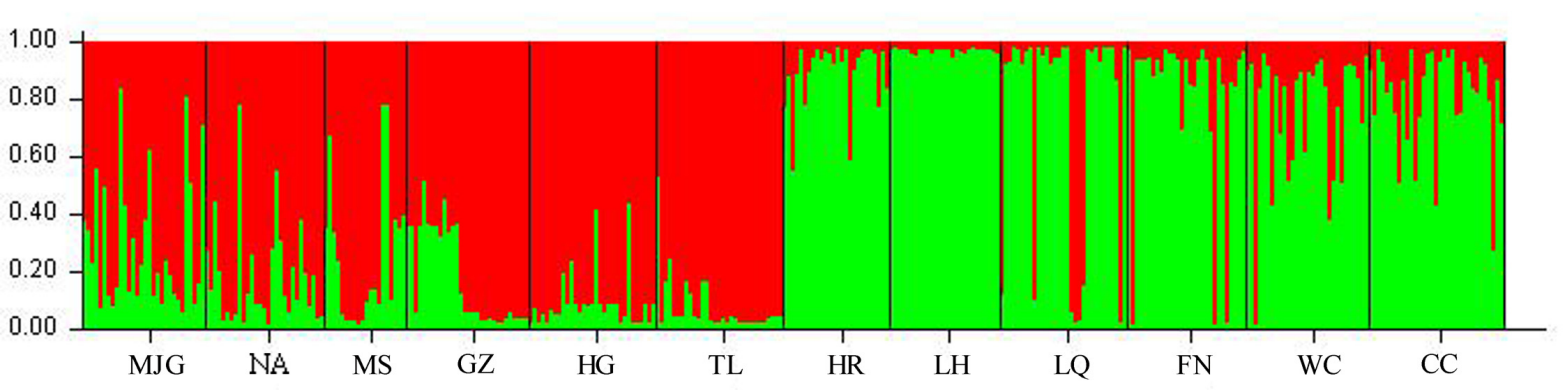
Bayesian STRUCTURE bar polt of membership for 12 *C*. *mandshurica* populations (K = 2). Red represents GroupsⅠand green represents GroupsⅡ. For details of locations abbreviations and locations, see Table 1 and Fig 1.

The 348 hazelnut individuals were further assessed for population stratification using the STRUCTURE program. SSR data were analyzed with possible cluster number (K-value) ranging from 1 to 12. The ΔK showed a clear maximum for K = 2 (ΔK = 185.0287), indicating among 12 populations the existence of two groups (S1 Fig). Group I included populations from MJG, NA, MS, TL, HG and GZ. Group II included populations from HR, LH, LQ, FN, WC and CC (Fig 3). Structure analysis suggested differentiation between the two groups, and grouped them approximately in line with the geographic area. These results indicated that there were different degrees of introgression in the populations, detected as differences in allele frequencies among the populations.

**Fig 4:**
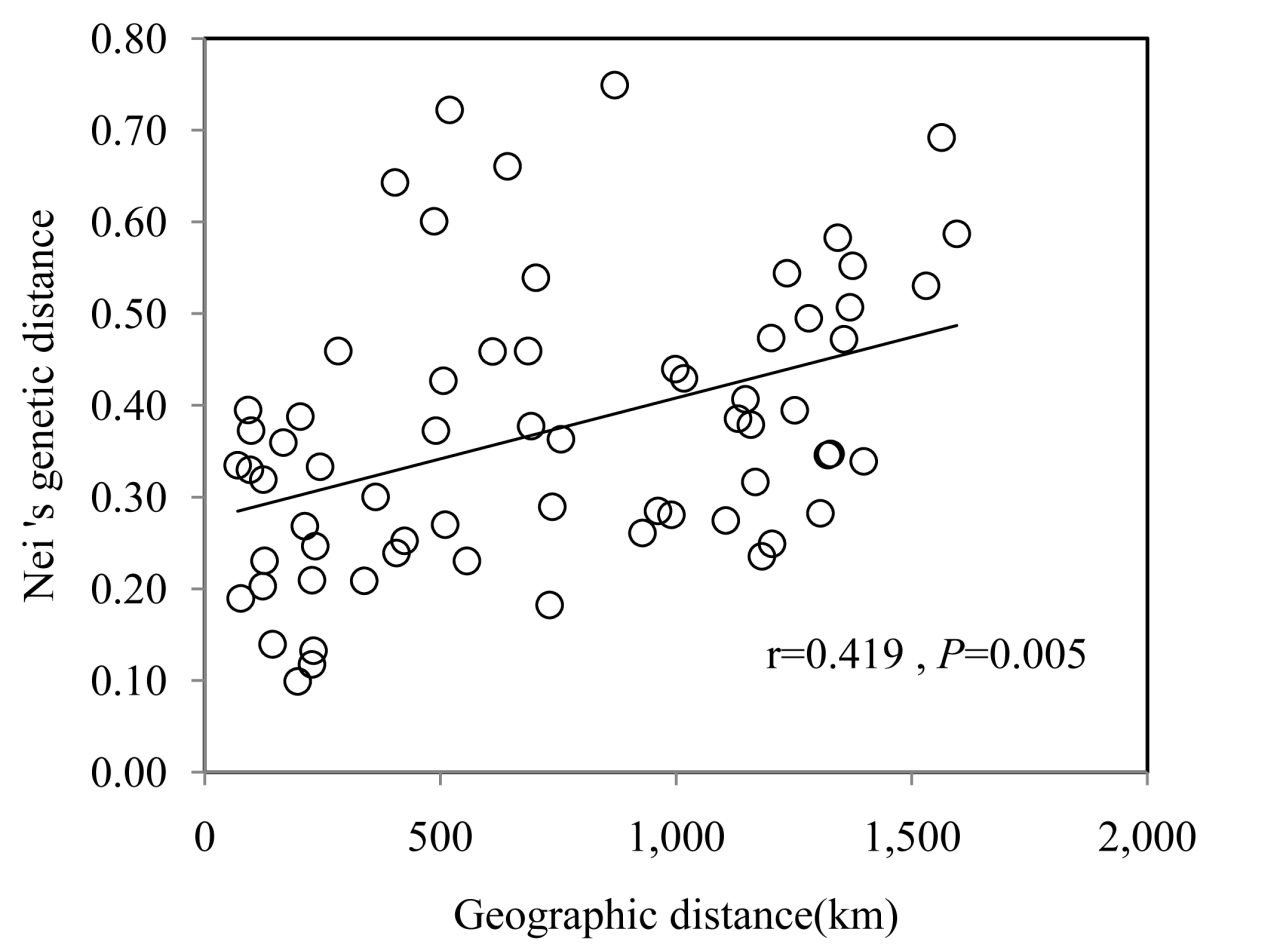
Mantel test for matrix correlation between Nei’s genetic distance and geographic distance for 12 *C*. *mandshurica* populations.

The Mantel test revealed a weak but positive correlation between Nei’s genetic distance and geographic distance (km) (r = 0.419, *P* = 0.005; Fig 4), suggesting that genetic differentiation in the 12 populations might be caused by geographic isolation due to distance.

## Discussion

### SSR polymorphism and genetic diversity among populations

In recent years, SSR marker techniques have been extensively applied in the detection of genetic variation in hazelnut populations, thereby estimating their genetic diversity [34–39]. In this study, the SSR marker analysis showed a high degree of genetic diversity *C*. *mandshurica*. The average number of alleles observed per locus (15.3) was significantly higher than other *Corylus* species such as *C*. *heterophylla* (4.18) [40] and *C*. *avellana* (7.16 [10], 7.1 [41] and 11.9 [25]. *Ho* and *He* values were 0.6709 and 0.7954 respectively. These results were similar to the values previously reported by Bassil *et al* [10] for *C*. *avellana* and Wang *et al* [40] for *C*. *heterophylla*.

The high genetic diversity in *C*. *mandshurica* populations may relate to the biological characteristics and living environment of this species. *C*. *mandshurica*, as a widely distributed, perennial woody plant species, can preserve its genetic diversity for quite a long period of time. Moreover, *C*. *mandshurica* is monoecious, wind-pollinated and self-incompatible mating system of species. In a long term, outcrossing will decrease differentiation among individuals in a diverse population. In addition, *C*. *mandshurica* is an ancient species capable of clonal propagation due to the formation of adventitious buds on rhizomes. Asexual reproduction and sexual reproduction of plants will produce a life-long effect. Clonal and sexual preproduction can result in many generations coexisting in a population. Such populations are less susceptible to genetic drift [42] and help to preserve genetic variation.

### Genetic structure, gene flow and differentiation among populations

AMOVA revealed that 11.50% of variation is among populations and the remaining 88.50% of variation is within populations (P<0.001) (Table 4). Moreover, the results of AMOVA analysis were consistent with the mean value of *F*_*st*_, further suggesting the existence of low genetic differentiation among different populations. Similar results were recently reported by Di *et al* [38], who analyzed 8 populations of *C*. *heterophylla* and showed that 16% of the genetic variation existing among populations.

Comparison of these results with the UPGMA dendrogram and the Bayesian STRUCTURE (K = 2) bar plot showed general agreement between the population subdivisions and the genetic relationships among the populations. The populations were divided into two main clusters. Similar results were previously reported on Chinese walnut (*Juglans mandshurica*) [43], two refuges in the last ice age have been inferred: The Taihang and Qinling Mountain in North China and Changbai Mountain in Northeast China. Another possible reason relates to the existence of the Yanshan Mountains. It is worth noting that a number of genetically similar populations were located in the same geographic region. The Mantel test revealed a significant correlation between the genetic distance and geographic distance among populations (r = 0.419, *P* = 0.005). Therefore, it’s speculated that the genetic structure of *C*. *mandshurica* population in China can be affected by geographic distance.

Gene flow is defined as the transfer of alleles from one population of a species to another. Study of gene flow is critical for the understanding of population processes within and among species [44]. The carrying of alleles to a population where they did not previously exist can be a very important origin of genetic variation [45]. The most important factor impacting the rate of gene flow between different populations is mobility [46]. Slatkin [47] considered that if *Nm*>1, the gene exchange among population can prevent the impact of genetic drift and reduce the genetic variance among populations. In this study, the average of gene flow of *C*. *mandshurica* at each locus was 1.8080, indicating that the gene flow was one of important factors influencing the genetic structure of *C*. *mandshurica* populations. The relatively high level of gene flow likely prevents genetic differentiation, which is the reason for the observed low genetic differentiation. That is the reason why the variance within populations was significantly higher than that between populations. Therefore, the lack of differentiation is attributed to gene flow.

Gene flow among populations was influenced by a number of factors including the mating system, geographic distribution, mechanisms of seed dispersal, and the stage of succession and colonization [48, 49]. An earlier study found that the pollen and seed dispersal was the foremost mechanism for gene exchange among plants [50]. As pointed out in a more recent study, gene flow mainly arises from two diffusion processes: seed, the extent and relative importance of which largely influence the landscape of neutral genetic diversity across a geographical area [44]. *C*. *mandshurica* is wind-pollinated and self-incompatible; its pollen is easily dispersed over medium and long distances. In addition, the nuts of *C*. *mandshurica* are nutritious and thus serve as an important food source for small mammals and birds, who can transport them in small numbers over long distances. All of these factors can contribute to the observed high gene flow between different populations.

### Genetic resource conservation strategies

In recent years, it has become increasingly important to adopt a holistic view of biodiversity, which includes agricultural biodiversity, conservation and sustainable development. An in-depth understanding of hazelnut genetic diversity and ecological distribution is indispensable for the protection and utilization of this species [35]. Analyzing the genetic diversity and genetic structure of *C*. *mandshurica* populations can provide a theoretical basis for the conservationist to formulate future conservation strategies of *C*. *mandshurica*.

In this work, the results based on SSR techniques discovered that wild *C*. *mandshurica* populations have a relatively high level of genetic diversity. However, in the past several decades, excessive deforestation and over-exploitation have damaged the ecological environment of many wild *C*. *mandshurica* living on natural hills. And local farmers have devastated the natural habitats of some populations in order to obtain the economic value. If not properly preserved, this species will likely become endangered because of both high demand and adverse climatic conditions, and even become extinct in the future. We suggest *in situ* conservation method: conservation to prevent the decrease in population sizes and loss of genetic diversity. Moreover, in order to achieve effective conservation of *C*. *mandshurica* germplasm resources, efforts are needed to carefully plan and construct pollen banks and gene banks for hazelnut. In the meantime, protected areas can be established to conserve and restore the habitat and the populations. In addition, it is important to develop a core collection of *C*. *mandshurica* in greater breadth and depth, which would not only mitigate the pressure of excessive use of wild resources, but also help achieve more effective management and use of hazelnut germplasm.

## Supporting Information

S1 Fig Delta K value as a function of K based on 10 runs, indicating the most likely number of two genetic clusters. DeltaK = mean (|L''(K)|) / sd(L(K)).
(TIF)

S1 Table Nei's genetic distance among *C*. *mandshurica* populations. (DOCX)
