# Supplementary figures and images for: Assessment of Genetic Diversity and Population Genetic Structure of Corylus mandshurica in China Using SSR Markers
Source: PLoS One. 2015 Sep 10;10(9):e0137528. doi: 10.1371/journal.pone.0137528 (PMC4565687; doi:10.1371/journal.pone.0137528)

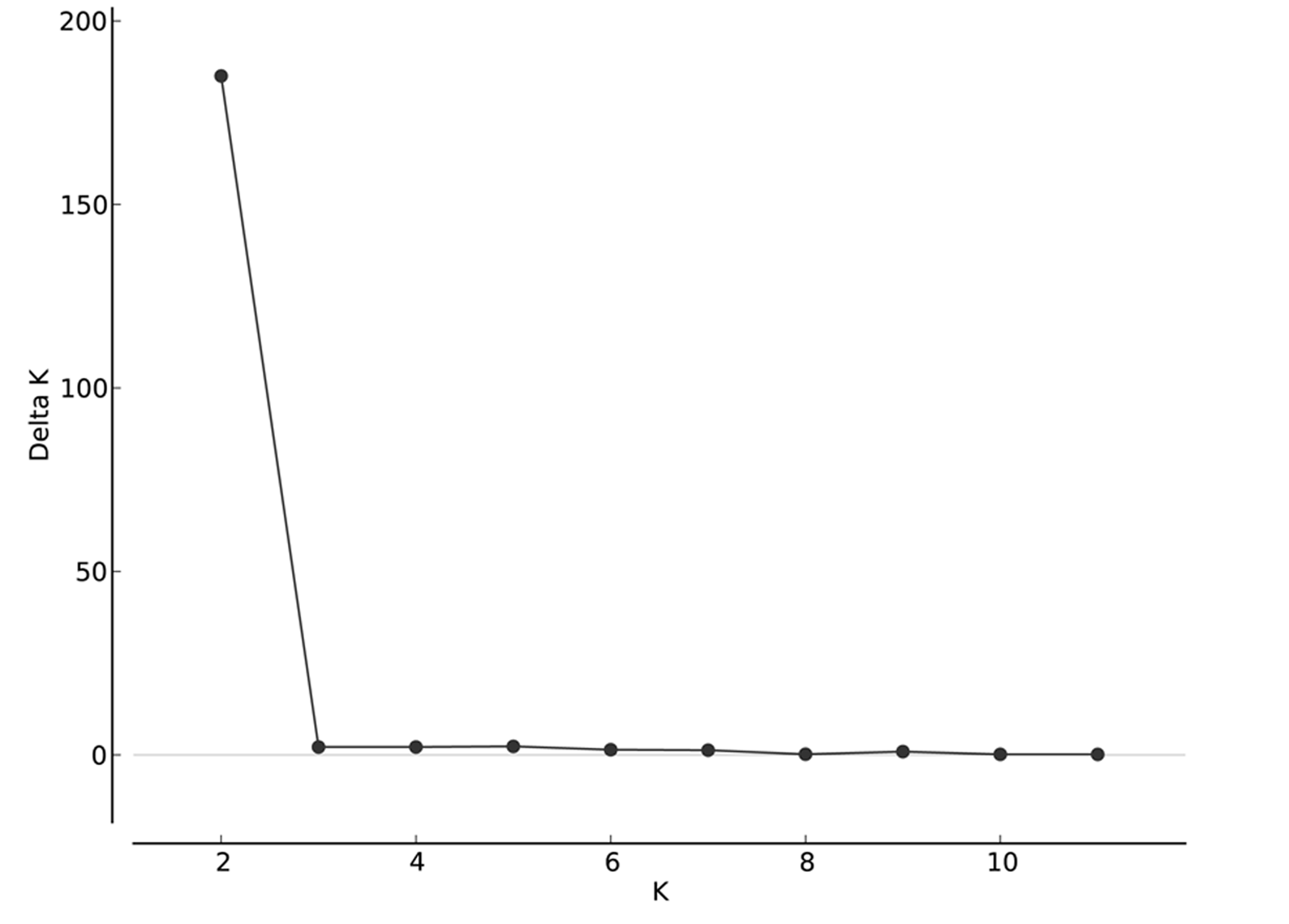

Supplement: S1 Fig — DeltaK = mean (|L''(K)|) / sd(L(K)). (TIF) [file pone.0137528.s001.tif]
