# Supplementary material for: Assessment of Genetic Diversity and Population Genetic Structure of Corylus mandshurica in China Using SSR Markers
Source: PLoS One. 2015 Sep 10;10(9):e0137528. doi: 10.1371/journal.pone.0137528 (PMC4565687; doi:10.1371/journal.pone.0137528)
Supplement: S1 Table — (DOCX) [file pone.0137528.s002.docx]

|  | MJG | HR | LH | LQ | FN | NA | MS | WC | TL | CC | HG | GZ |
| --- | --- | --- | --- | --- | --- | --- | --- | --- | --- | --- | --- | --- |
| MJG | —— |  |  |  |  |  |  |  |  |  |  |  |
| HR | 0.2822 | —— |  |  |  |  |  |  |  |  |  |  |
| LH | 0.3166 | 0.1396 | —— |  |  |  |  |  |  |  |  |  |
| LQ | 0.5304 | 0.2094 | 0.3001 | —— |  |  |  |  |  |  |  |  |
| FN | 0.3946 | 0.3725 | 0.3950 | 0.4592 | —— |  |  |  |  |  |  |  |
| NA | 0.1177 | 0.4065 | 0.4293 | 0.5521 | 0.2746 | —— |  |  |  |  |  |  |
| MS | 0.2305 | 0.5070 | 0.5441 | 0.5868 | 0.3458 | 0.1325 | —— |  |  |  |  |  |
| WC | 0.3855 | 0.3878 | 0.3347 | 0.2389 | 0.2029 | 0.2806 | 0.2492 | —— |  |  |  |  |
| TL | 0.3773 | 0.6606 | 0.7221 | 0.7490 | 0.4587 | 0.2700 | 0.2895 | 0.4268 | —— |  |  |  |
| CC | 0.3472 | 0.3297 | 0.3595 | 0.2682 | 0.1895 | 0.2355 | 0.3388 | 0.0991 | 0.4592 | —— |  |  |
| HG | 0.3192 | 0.5826 | 0.4733 | 0.6919 | 0.4948 | 0.2087 | 0.2466 | 0.3791 | 0.3631 | 0.4720 | —— |  |
| GZ | 0.2607 | 0.6006 | 0.6430 | 0.5391 | 0.3724 | 0.1823 | 0.2848 | 0.2524 | 0.3330 | 0.2304 | 0.4396 | —— |
|  | | | | | | | | |  |  |  |  |

**S1 Table. Nei's genetic distance among *C.mandshurica* populations**
